# Methods to identify, study and understand End-user participation in HIT development

**DOI:** 10.1186/1472-6947-11-57

**Published:** 2011-09-28

**Authors:** Anna Marie Høstgaard, Pernille Bertelsen, Christian Nøhr

**Affiliations:** 1 Department of Development and Planning, Virtual Centre of Health Informatics, Aalborg University, Fibigerstræde 13, 9220 Aalborg Ø, Denmark

## Abstract

**Background:**

Experience has shown that for new health-information-technology (HIT) to be suc-cessful clinicians must obtain *positive clinical benefits *as a result of its implementation and *joint-ownership *of the decisions made during the development process. A prerequisite for achieving both success criteria is *real *end-user-participation. Experience has also shown that further research into developing improved methods to collect more detailed information on social groups participating in HIT development is needed in order to support, facilitate and improve real end-user participation.

**Methods:**

A case study of an EHR planning-process in a Danish county from October 2003 until April 2006 was conducted using process-analysis. Three social groups (physicians, IT-professionals and administrators) were identified and studied in the *local, present *perspective. In order to understand the interactions between the three groups, the *national, historic *perspective was included through a literature-study. Data were collected through observations, interviews, insight gathered from documents and relevant literature.

**Results:**

In the local, present perspective, the administrator's strategy for the EHR planning process meant that there was no clinical workload-reduction. This was seen as one of the main barriers to the physicians to achieving real influence. In the national, historic perspective, physicians and administrators have had/have different perceptions of the purpose of the patient record and they have both struggled to influence this definition. To date, the administrators have won the battle. This explains the conditions made available for the physicians' participation in this case, which led to their role being reduced to that of clinical consultants - rather than real participants.

**Conclusion:**

In HIT-development the interests of and the balance of power between the different social groups involved are decisive in determining whether or not the end-users become real participants in the development process. Real end-user-participation is essential for the successful outcome of the process. By combining and developing existing theories and methods, this paper presents an improved method to collect more detailed information on social groups participating in HIT-development and their interaction during the development. This allows HIT management to explore new avenues during the HIT development process in order to support, facilitate and improve real end-user participation.

## Background

The key objectives for healthcare services in all counties are high patient safety and high quality of treatment and care. A significant factor in achieving these objectives is an optimally functioning information and communication infrastructure, which ensures that the right information is communicated at the right time and place to the right persons. Over the past approximately 50 years, booming healthcare and technological development has meant that the paper-based health record, which for decades has provided the infrastructure within the healthcare sector, no longer meets these requirements. Concurrent advances in information technology (IT) indicate that an electronic-based health record can resolve many of the problems associated with the paper-based health record, e.g. accessibility and data validity. This has caused many healthcare providers to make great efforts to replace the paper based-health record with an electronic record.

Furthermore in Denmark - where the healthcare system is public and financed by taxation and the five regions^1 ^govern the hospitals - there has been extensive discussion about the EHR in Danish hospitals. Since 1999, the Danish national strategy for IT in the healthcare sector has required that all Danish counties implement an EHR [1]. In Denmark the concept of "EHR" is defined as a platform with different modules delivered by different vendors. This means that the EHR in the five Danish regions each have their own development strategy and different platforms.

The national strategy for IT resulted in the County of North Jutland (CNJ) developing an overall IT-strategy for the EHR development process [2]. In March 2004, a local EHR working group was established with a view to producing the requirement specifications for the EHR and choosing between four possible systems.

Studies have shown that the introduction of new HIT systems - including the EHR - besides solving some problems, often brings with it a number of new problems, including some of an organizational nature [3-15]. However, research has also revealed not only pitfalls to be aware of but also success factors to be met in order for an EHR implementation to be successful [14,16-20], i.e. that the clinicians must obtain *positive clinical benefits *[14,15,21] as a result of an EHR implementation and *joint ownership *[4,5,22-24] of the decisions made during the development process. A prerequisite for both is real participation in the EHR development process enabling clinician's to exert real influence in decision-making [4,5,16,22,24-27]. Mumford classifies user-participation in IT-development in three different types according to depth: consultative, representative and consensus participation [28,29]. According to Mumford *real *influence - not pretended or symbolic - in HIT-development is best achieved by using the consensus type of participation, where the users are involved *throughout *the technological development process [28,29].

Prerequisites for real participation/influence are as follows.

• *Early *involvement of clinicians (end-users) in HIT development is essential because, as the technological development process proceeds, the scope of influence of the clinicians and the possibility of changing the decisions already made are progressively limited [22,23,30-32].

• The best possible *representation *of all groups of clinicians in order to achieve joint ownership from all relevant actor-groups [25,28,33]).

• The possibility of *workload reduction *- meaning that colleagues take over part/all of the clinical duties for the clinicians involved (it does not involve extra payment) [22,26].

When it comes to achieving a successful implementation of the EHR, all groups of clinicians are important user participation groups in the development process. However, more studies show that physicians are a very important group because their acceptance is crucial as to whether or not the EHR is implemented in the intended way [34-36].

Based on a literature review and the results of an AMIA workshop, Kaplan et al. [17] report that many HIT systems are not successful "despite an accumulation of best practices research identifying success factors". Kaplan et al. therefore call for further research into the development of improved methods for successful HIT development. Even though user involvement/user participation is known to be a very important success factor, no empirical studies of user involvement in the *health information *field have been conducted in recent years, whereas a number of studies of user involvement have been completed in the *medical device *field [37-41].

In this perspective, an improved method for collecting more detailed information on end-user participation in HIT development in order to support, facilitate and improve *real *end-user participation was developed throughout a research study of the EHR planning process in the County of North Jutland (CNJ) focusing specifically on *physicians *as a relevant social group in EHR development. Besides the physicians, IT-professionals and administrators were identified as significant groups based on prior research and own experiences [14,35,36,42].

The objective of the research was to develop an improved method for identifying, studying and understanding end-user participation in HIT development in order to collect more detailed information on social groups participating in HIT development and their interaction during the development. This allows HIT management to explore new avenues during the HIT development process in order to support, facilitate and improve real end-user participation.

The objective was achieved by answering the following two research questions:

1. Did the physicians have the necessary resources (interest, power, organization, information, access and knowledge) during the EHR planning process to change their status from potential to actual social carriers^2 ^of the EHR-technology?

2. Can the answer to the first research question be understood by studying the different "meanings"^3 ^that each of the relevant social groups, (physicians, IT-professionals and administrators), attached/attaches to the EHR?

An improved method to collect more detailed information on social groups participating in HIT development and their interaction during the development will fill a void in the current body of knowledge about success factors and best practices and, not least of all, on how to apply these in practice.

## Theoretical framework

The new methodological approach is based on the combination and further development of the following two theories:

• the Socio-Technical-Carrier-of-Technology theory (STCT)

• the Social Construction of Technology theory (SCOT).

The STCT theory was developed by researchers at Aalborg University in the 1990s by combining and further developing two theories: the Socio-Technical theory and the Social-Carrier-of-Technology theory.

### The Socio-Technical theory

In the Socio-Technical theory a broad concept of technology is introduced, focusing on the micro-level and the actor as opposed to the macro-level. This technology concept is open-ended to enable an understanding of the relation between technological and social change. According to the concept, technology embraces a combination of four constituents: technique (meaning the technological object in question), knowledge, organization and product. These four constituents are inseparable components of any technology. A qualitative change in any one of the components will eventually result in supplementary, compensatory, and/or retaliatory change in the other components. For a technology to be considered as such, it has to be applied and result in a product, and for this to happen, actors have to be active within each of the four components.

**Figure 1:**
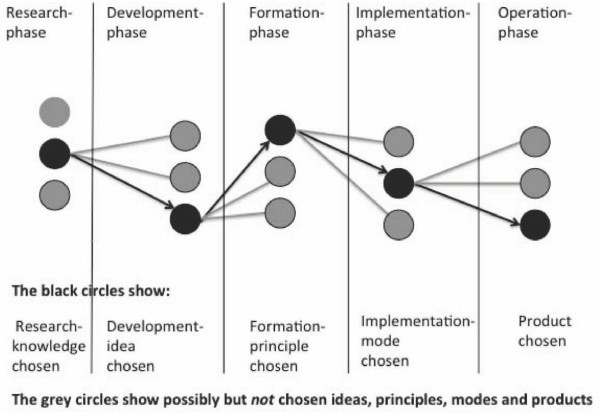
**The five stages in the technological development process**. Figure 1 shows that at every stage a selection takes place (black circles) which leaves out many potential actions not chosen (grey circles).

The Socio-Technical theory is process-oriented and focuses on the technological de-velopment process. It can be described in five stages. Within each of these stages, the actors make their selections based on possibilities and interests. This means that at every stage a selection takes place which leaves out many potential actions not chosen (Figure 1) [43-45].

### The Social-Carrier-of-Technology theory

The focus of the Social-Carrier-of-Technology theory is the actors. It was developed to identify and study the relevant "Social-Carriers-of-Technology" involved in the development of new technology^4^. The concept refers to a group of actors, or a social entity, who choose the new technology and carry it forward towards the next phase in a technological development process. According to this theory, this will happen only if the "Social-Carriers-of-Technology" have the necessary resources (interest, power, organization, information, access and knowledge) to change their status from *potential *to *actual *carriers of the technology in question. Often two or more Social-Carrier-of-Technology groups choose and implement the technology together. These groups are named *combined *Social-carriers-of-Technology. When, on the other hand, the social groups involved carry the technology in different phases of the development process and, in this way, are sequentially linked to each other, they are named *linked *Social-Carriers-of-Technology. In reality the combined and linked Social-Carriers-of-Technology are most often intertwined [46].

### The Socio-Technical-Carrier-of-Technology theory (STCT)

The theory developed by combining the Socio-Technical theory and Social-Carrier-of-Technology theory is termed: the Socio-Technical-Carrier-of-Technology theory (STCT) [26,43,47,48]. According to this theory, the choices made by the actors include not only the technique but all four components in the socio-technological technology concept: technique, knowledge, organization and product. The STCT theory states that every qualitative change - and the final outcome/product - in a technological development process can be traced back to a change in the composition of the social carriers of technology and in the conditions necessary to achieve the status of an actual Social-Carrier-of-Technology.

With a focus on physicians, the different social groups' participation in the EHR planning process and their influence in decision-making were studied by examining:

• the vested interests of the different relevant social carrier groups of technology in relation to the EHR (interest);

• their options to carry through these interests (power);

• the degree of support from their professional association (organization);

• their opportunities to see and test the system in practice (access); and

• the amount of knowledge and information they had acquired about EHR both in general and particularly regarding the different options available (knowledge and information)

In the present study the STCT theory was used as a framework to shed light on the interactions that occurred between the different relevant social groups in the *local, present *perspective - i.e. within a relatively short time frame.

### The Social Construction of Technology theory (SCOT)

According to the SCOT theory, every stage in a technological development process involves choices between various options. These choices are made by the different social groups involved based on the different "*meaning*"^5 ^they attach to the technological object in question. This means that the design and the further development of the technological object is as a result of gradual and mutual debates between the different social groups, and that besides rather narrow, purely professional considerations, social factors (interests, power) determine which options are chosen. This results in a "multidirectional" development model in contrast to the linear model often described in the history of technology [49].

During the technological development process different *problems *will appear as a result of the different "meaning" each social group attach to the technological object. These problems are resolved through negotiations between the groups - the final solution being highly dependent on the interests and the amount of power of each group involved [49].

The various social groups operate from different "*technological frames*". A technological frame represents e.g. the purposes, the goals and the problem-solving processes attached to the technological object - historically and nowadays - for the different social groups. The contents of this frame determine which "meaning" a particular social group attaches to the technological object, and it also structures the interaction between the actors within each group [49].

According to the SCOT theory, studies of a technological development process must start out by identifying the relevant social groups. It is obvious that the end-users represent a relevant social group, but also less obvious social groups must be identified. Once the relevant social groups are identified, the next step is to identify the different problems that each social group attach to the technological object. For each problem it is then possible to identify a range of solutions from the social groups involved. This way of describing the development process clearly shows the interpretive flexibility of the technological object and of its future functionality. It also clearly demonstrate that during a technological development process a number of conflicts will arise: different social groups express various technical needs, numerous solutions to a problem, moral conflicts, etc. [49].

In the present study the SCOT theory provided a framework for understanding the underlying reasons for the different social groups' vested interests in the health record and the power they had or did not have to bring forward those interests. The *historical *perspective was, therefore, included in an attempt to uncover the "meaning" that has been attributed by the different relevant social groups to the health record from its origins in a paper format to the electronic format of today - and in order to gain an understanding of the historical and current interests and the balance of power between the social groups.

The STST and the SCOT theories are both well-known. What is new is the act of combining them and employing them within the healthcare sector, which has not been done previously.

## Methods

### Design

In accordance with the two research questions the research project was divided into two parts: a case study and a literature study.

### Case study

The three Social-Carrier-of-Technology-groups (the STCT theory) were studied in the *local, present *perspective with a focus on process-orientated technology analysis. They consisted of physicians and IT-professionals in the EHR working group and the ICT-board of the CNJ. The context was the EHR planning process in the CNJ in Denmark, and the research period lasted from October 2003 until April 2006.

### Literature study

In the literature study the three relevant social groups (the SCOT theory) were studied in the *national, historic *perspective. They consisted of physicians and IT-professionals associated with the Danish hospital sector and hospital managers and EHR decision-makers at county/regional level.

### Data collection and analysis

Data in the case study (the local, present perspective) were collected as presented in Table 1.

**Table 1:** Data collection in the case study

Data collection techniques	Techniques used for this study
**Interviews**	Semi structured interviews with 11 physicians (including all physicians in the EHR working group), 2 IT-professionals, 2 Administrators. The interviews, lasting from 60 min., were recorded and transcribed.

**Observations**	Participant observation at EHR working group members

**Insight into documents**	Minutes from meetings, project plans, tender material etc.

**Validation of data**	Data-triangulation, transcripts sent to interview-persons, written report sent to participants

Data were analysed using a Socio-Technical "*technology-carrier analysis*" developed by researchers at Aalborg University [30,43,47]. The software programme ATLAS [50] was used to analyse interviews. The focus was on the six conditions required to achieve the status of an actual social carrier of EHR (interest, power, organization, information, access and knowledge) and an "open mind" towards other themes.

A new framework of visualizing the results of the technology-carrier analysis was developed (table 2). A number of marks were given to indicate whether the conditions to become an *actual *social carrier of the EHR were met for the different social groups involved.

**Table 2:** Framework for visualization the technology-carrier analysis.

Social group	Interest	Power	Organization	Information	Access	Knowledge
**Physicians**						

**IT-professionals**						

**IT-board**						

The results of the analysis will be added in the tablebody.

Three marks () indicate that the group achieved the status of an *accrual *social carrier of EHR. Two marks () indicate that the group was a *very potential *social carrier - and one () indicates that the group was a *less potential *carrier. It is important to stress that the table only serves as a visualization of the results; the number of marks given are based on the researchers *thorough *qualitative analy-sis of each of the six conditions involved.

In the literature study, data were collected through searches in PubMed and Google Scholar. The research strategy was as follows:

• the history and the development of the Danish health record - paper based as well as electronic based

• the history of the Danish physicians, their interest in the health record and the way in which they were/are organized

• the history of the Danish IT-professionals and the Danish administrators, their respective interests in the health record and the way in which they were/are organized

Also relevant textbooks were used within the above areas [51-53].

Data were analysed using SCOT analysis [49,54]. The analysis included:

• identifying the technological object and the relevant social groups and

• studying the different meaning that these groups attach to the technological object and their respective technological frames.

### Ethical approvals

According to Danish law ("Law on the Scientific Committee System and the treatment of biomedical research, chapter 3, section 3"), formal approval from "The Danish National Committee on Research Ethics" was not required. Concerning the individual participants (physicians, IT-professionals and administrators) informed consent was obtained prior to data collection.

## Results

### Technology-carrier analysis

#### The planning strategy: factors affecting all six conditions

Some factors related to the planning strategy affected all six conditions. They are reported in this section.

An EHR working group was established by the ICT-board to draw up requirement specifications and to choose between four EHR systems. The members of the working group comprised physicians, nurses, secretaries and IT-professionals. Two out of eight physicians in the EHR working group were members from the start. The other six joined the group 18 months later.

The - predominantly informal - planning strategy for the EHR planning process meant that there was no clinical workload reduction at all during the process. Physicians were expected to handle their full-time clinical duties, while at the same time participating in the planning process. They were expected to read a vast number of ICT- technical papers and reports. Consequently, seven out of eight physicians in the working-group were senior physicians from Aalborg Hospital (the main hospital in the CNJ) who to some extend mastered their clinical tasks. Only one junior physician - from a hospital outside Aalborg - joined the group. He participated in two meetings whereupon he had to leave the group due to lack of time. It meant that junior physicians (more than half of the physicians in the CNJ) and physicians from hospitals outside Aalborg were not represented in the "EHR Working Group".

During the planning process the EHR-project management made no attempts to learn from the experiences of management of EHR planning-processes in the other Danish counties - including experiences about the need for workload reductions in one form or another. The EHR-project management's main argument for not allowing workload reduction (paid by the county) was a general principle about leaving decisions about workload reduction to the individual hospitals in the county. From the ICT-board' point of view, the local hospital's incentive to pay for workload reduction was the fact that the individual hospitals were allowed to keep any rationalization gains from the EHR implementation process themselves. This principle meant that the responsibility for prioritizing costs for workload reduction e.g. treatment and care, was moved from county-level to hospital-level. Based on experiences regarding the prerequisites for a successful implementation of EHR, a decision like this should be made at county-level to ensure the best possible implementation across the county. The fact that no sharing of past experiences at any level (strategic, tactical and operational) took place before or during the EHR planning process could indicate that the EHR-project management had underestimated the workload associated with the planning process.

#### Interest

The three technology-carrier groups had different interests in the implementation of the EHR. The physicians' main interest was to ensure positive clinical benefits. To achieve this, physicians found it important that it was physicians, who formulated the medical demands in the requirement specification. The IT-professionals' main interests were concerned with optimizing administrative functions in the EHR, while the ICT-boards had a major interest in complying with the national requirements about implementing EHR in all Danish counties.

A new way of implementing the planning process was used in the CNJ: a "dialogue based planning-process". It facilitated dialogue between the members of the working group and the four vendors possible, and it made it possibly for each of the vendors to change system functions - e.g. the configuration of the user-interface - during the process. The physicians felt that this enabled them to gain some insight into more aspects of the systems and to argue for their clinical demands and interests in the EHR. However, the physicians did not have sufficient time (no workload reduction) to go through all documents related to the process. Neither did they have the time to participate in all meetings in the "EHR Working Group" or meetings and workshops in the sub-groups established in relation to the process. As a consequence, IT-professionals with past clinical background (non-physicians) developed most of the medical demands in the requirement specifications. This implies according to Kensing et al. [25], Brandt [55] and Simonsen et al. [56] a great risk; namely that the medical requirements do not reflect the clinical reality, because experience shows that professional knowledge has to be presented by the professionals. At the same time, it implies a great risk in that the physicians' interests were not, or only partially, met during the process.

All four EHR systems were at a very early developmental stage at the time of the planning process. None of them were in operation in any hospital ward - they only existed as the vendors schematic diagrams and early prototypes. This fact taken together with the fact that all the vendors changed the system functions related to clinical work practices (e.g. configuration of the user interface, the number of mouse clicks and integration with other systems) during the development process, resulted in the four systems becoming almost alike. Therefore clinical related functions were abandoned as criteria for selection of the EHR system. Now only technical differences were used to choose between the systems. Thus, the physicians made their choice according to feelings, sensations - and advice from the IT-professionals. Thus, the final choice of EHR system was primarily made on the basis of the interests of the IT-professionals and the ICT-board, i.e. according to technical and economic criteria.

#### Power and organization

The Danish Medical Association was not involved in the planning process in any way. The organizational support and encouragement that the physicians in the working group achieved during the planning process came from colleagues at Aalborg Hospital.

Compared to the physicians, the IT-professionals had much more organizational support - and power. The fact, that their organization was in charge of the project management and - compared to the physicians - they were well represented in both the ICT-board and all other groups related to the process - and that most of the documents prepared during the EHR process were prepared by members of their organization - gave them a significant amount of power.

**Figure 2:**
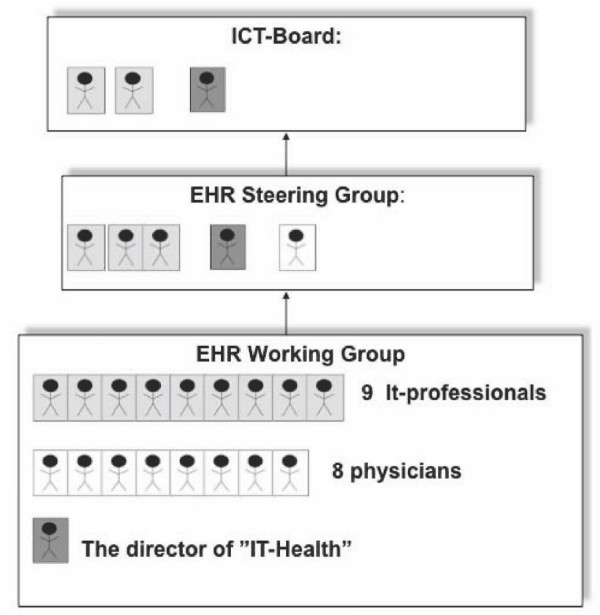
**Persons in the three technology carrier groups**. Figure 2 shows that the director of IT-Health and two of the IT-professionals were members of both the EHR Working Group, the EHR Steering Group and the ICT-board, while no physicians were member of the ICT-board and only one physician was a member of the EHR Steering Group.

The director of IT-Health was also the EHR-project manager. At the same time, the director and two heads of department at "IT-Health" were members of both the ICT-board and the EHR Steering Group. Thus, the IT-professionals were part of the decision-making authority (Figure 2).

The physicians and the IT-professionals also used the power associated with profes-sional knowledge to accommodate their own respective interests. However, the physicians' clinical knowledge turned out to be inadequate as a basis for choosing between systems. Technical knowledge was essential.

The ICT-board had the power to make final decisions - which they did, when they ordered all physicians to choose one of the systems despite the physicians' request to wait until more useful information, access and knowledge were available. In fact, the physicians were powerless when it came to the possibility of being able to exert real influence on which system to choose.

#### Access

The four systems selected were not in operation in any hospital ward and therefore, it was not possible to test them in a "real live setting". This was critical, especially to the physicians as they were asked to specifically concentrate on the system functions related to clinical work practices (e.g. configuration of the user interface, the number of mouse clicks and integration with other systems). Recognizing this problem, the physicians were offered to test four prototypes. None of the physicians were able to participate in *all *four tests because of clinical duties (no workload reduction), which meant that they were unable to compare the four systems. Thus, the tests did not provide the physician with a better basis for choosing between the systems. The fact that the four EHR systems could not be tested in a real live setting meant that clinical knowledge was not enough to chose between the systems. Technical knowledge became essential (see the knowledge aspect).

#### Information

The members of the working group, who were involved from the start, were invited to different arrangements as preparation for the different tasks they were asked to perform. The six physicians, who joined the working group 18 months after it was established, did not receive any kind of introduction or update on the work conducted so far - or any other kind of preparation for the task. This fact together with the fact that clinical workload reduction was not possibly - meaning that the physicians had their full-time clinical work beside the work in the EHR-working group - made it impossible for the six last physician members of the group to obtain the level of information necessary to achieve real influence in decision-making.

#### Knowledge

Because the four systems became almost alike with respect to system functions re-lated to clinical work practices, these were given up as criteria for selection between systems. This meant that clinical knowledge became inadequate as a basis for choosing between systems - technical knowledge was essential. Only one of the eight physicians had the necessary technical knowledge for true participation in the often very technical debates in the working group. The old saying: "knowledge is power" proved to be very true. The reality was: the more technical knowledge - the more power. The lack of technical knowledge made the physicians incapable of exerting real influence on several important decisions made during the process - e.g. the final choice of EHR system.

#### Synthesis of the technology carrier analysis

The analysis showed absence of workload reduction as the main barrier to the physicians to achieve true involvement in the process, and it affected all six conditions - interest, power, organization, information, access and knowledge - required to obtain the status of actual social carriers of the EHR. The clinicians role in the process was reduced to clinical consultants informing about physicians needs in the requirement specifications and other documents. However, they were not able to fill this role completely due to lack of time. Therefore, this work was to a large extent handled by IT-professionals from "IT-Health". The answer to the first research question is that none of the conditions required in order to obtain the status of actual social carriers of the EHR were met for the physicians in the EHR working group. Their initial status as potential carriers of the EHR remained unchanged. The results of the technology carrier analysis are synthesized and visualized in table 3.

**Table 3:** Presentation of the results of the technology-carrier analysis

Social group	Interest	Power	Organization	Information	Access	Knowledge
**Physicians**					-	

**IT-professionals**					-	

**IT-board**					-	

Table 3 shows to which degree the conditions for becoming an actual social carrier of the EHR were fulfilled for the different social groups on a three point subjective scale. Three marks indicate full achievement.

### SCOT analysis

#### The technological object and the social groups

The technological object was the health record - paper-based or electronic. The social groups comprised physicians and IT-professionals associated with the Danish hospital sector and hospital managers and EHR decision-makers at county/regional level.

#### Meanings and technological frames^6^

Danish physicians form an inhomogeneous group with respect to their opinion on both problems and solutions associated with the paper-based health record. Therefore they were divided into two groups by the researcher: "*Clinical physicians*" and "*Early adopters*". Throughout the history of the health record the two groups of physicians have had an *internal *struggle for the power and the right to define the purpose of the health record. Throughout history, the "Early adopters" have advocated for the introduction of new versions based on visions of future clinical benefits - primary (clinical work) as well as secondary (teaching and research). The "Clinical physicians" have tried to "arrest" this development, partly because they have felt no need for new versions, partly in order to "keep up" with daily clinical practice. However, in the long-term perspective the "Clinical physicians" have always had to accept new - increasingly standardized - versions of the health record.

In the short-term perspective, however, several examples show, that the "Clinical physicians" have succeeded in curbing the development for a time [34,57,58]. The two groups of physicians have, however, recently taken a common external position when it comes to the primary purpose of patient data due to a growing interest in the use of health data for secondary non-clinical purposes (management and governance) among administrators and IT-professionals. This primary purpose is clinical use in daily practice. Secondary clinical use - and other secondary purposes - must not compromise the primary use.

Besides the internal power struggle about the right to define the purpose of the health record, the Danish physicians have also faced an *external *struggle against administrators, which has taken place ever since the outset of the Danish health record approximately 150 years ago. Historically, administrators have shown a growing interest in health records, because better possibilities for extracting data for primary and secondary clinical purposes also meant better possibilities for extracting data for secondary non-clinical purposes. The development of the EHR was originally initiated by the "Early adopters" with the *internal *control of treatment quality as its objective. This has, over the years, been overtaken by administrators with *external *control of quality, efficiency and financing as its objective. At the same time, the argument about patient safety, which for many years was used solely by physicians as an argument for using patient data for *clinical *purposes, is now also used by administrators to legitimize the use of patient data for *non-clinical *purposes. In recent years, the "Early adopters" - and thus the medical profession - have lost most of the influence on the development of the health record to the administrators, and the physicians' power and right to define the purpose of health records appears more diminished today than ever before.

#### Synthesis of the SCOT analysis

The SCOT analysis contributed to a deeper understanding of the underlying reasons for the physicians not to obtain the status of actual social carriers of the EHR by uncovering the different meanings attached to the health record by the different relevant social groups. The answer to the second research question is that the different meanings the three social groups attached to the EHR are rooted in an inherited balance of power between physicians and administrators specifically. Ever since the outset of the Danish patient record, clinicians and administrators have fought for the power and the right to define its purpose. So far, administrators have so far won this battle and seem to have a stronger position today than ever. This inherited battle of power was the major reason for the approach chosen for the planning process in the CNJ. This battle is considered to be the reason for the conditions made available to the EHR working-group by the ICT-board during the planning process; conditions that consequently reduced the role of physicians in the planning process in North Jutland to one of clinical consultants - rather than real participants.

## Discussion

### Relating the method presented to other approaches

While the method presented in this paper has been developed for the *healthcare sector *specifically and to cover the *entire *IT development process, most other methods for supporting and improving end-user participation in IT development have focused on the design stage [22] and/or have been developed for other organizations than the healthcare sector [22,25,29].

### Strengths and weaknesses of the study

The purpose of this research was to develop an improved method to identify, study and understand end-user (physician) participation in HIT development in order to support and facilitate *real *end-user participation in HIT development. Well-known prerequisites for real end-user participation are *early involvement *([22,23,30-32], *representation *[25,28,33] and *workload reduction *[25,26]. The new approach presented in this paper have shed light on the interactions that occurred between the different social groups involved in the EHR planning process in the CNJ and have provided an understanding of the underlying reasons for these. Through a thorough examination of the six conditions necessary to require the status of an actual carrier of EHR (interest, power, organization, information, access and knowledge) the interac-tions between the social groups involved in the EHR planning process in the CNJ were disclosed, while the SCOT analysis shed light on the underlying reasons for this. However, these analyses also uncovered the prerequisites for real participation (early involvement, representation and workload reduction) and revealed that they were not met during the process.

On the basis of our findings we argue that the six conditions necessary to require the status of an actual carrier of EHR are good analytical markers for whether the preconditions for real participation are met or not in HIT development. Thus the method demonstrated has proven effective as a tool to support, facilitate and improve real end-user participation.

The present study is a qualitative study using triangulation of different data collecting methods to validate the data. The analytical framework supports and strengthens data analysis as it is based on an integration of well-known methods,

The method was developed throughout a research study in a major Danish healthcare organisation. However, research shows that the interactions and the battle of power between different social groups are not specifically Danish phenomena, they can be found in HIT development in general [59]. Thus, we argue that a thorough study of the six conditions needed for the end-users to require the status of active carriers of a new technology, and thus for them to be real participants, will also in a broader context provide HIT management with valuable information on the social groups participating in HIT development and for the reasons for the variations.

The method was developed and used during *the planning *stage. However, we argue that it can be used at any stage as the importance of acquiring knowledge about and understanding the social interactions occurring between the social groups involved is equally important during all stages of HIT develoment.

For the method to be employed it is a precondition that management at all levels are actively supporting it throughout the process. This means that they must provide the resources necessary in terms of time and personnel, as the method could be rather time consuming, depending on which and how many emperically data collection-methods are used.

In qualitative studies there is a risk that the researcher has a predetermined opinion on the subject in question. It is also a risk that the researcher is "seduced" by the position taken by one group. To account for this, all activities throughout the process have been thoroughly described (transparency). The fact that one of the researchers (AMH) has participated throughout the planning process in all meetings, and has had access to most documents - electronic as well as paper-based - has made it possible to assess the truthfulness of, e.g. time pressure and the amount of documents related to the process.

## Conclusions

Real end-user participation is essential for the successful outcome of HIT development - and thus for fulfilling the key objectives for healthcare. However, the interests of and the balance of power between the different social groups involved in HIT development are decisive for real end-user participation.

The method presented in this paper is a new, improved methodological approach which has proven effective for collecting more detailed information on end-users participation in HIT-development by disclosing the social interactions that occur between the social groups involved. Providing an understanding of the underlying reasons for the variations in the social groups participating by uncovering the different meanings attached to the HIT in question by the different relevant social groups has also proven effective.

This improved method for disclosing and understanding the social interactions between social groups in HIT development provides an important tool for HIT project management at any level. It allows new avenues to be explored during the process in order to support, facilitate and improve real end-user participation.

## Competing interests

The authors declare that they have no competing interests.

## Authors' contributions

All of the authors contributed to the conceptual design of this study. AMH carried out the data collection, performed the analyses and was responsible for writing the manuscript. PB and CN were involved in drafting the manuscript and revising it critically for important intellectual content. All three authors have read and approved the final manuscript.

## Endnotes

^1 ^Jan.1. 2007, 13 Danish counties were merged into five regions

^2 ^A group of actors or a social entity, which chooses the new technology and carries it forward towards the next phase in the process.

^3 ^Significance, goals, interests, needs (a concept used in the SCOT theory).

^4 ^In the Social-carrier-of-Technology theory the concept "technology" means technique.

^5 ^Purpose, significance, goals, interests.

^6 ^Includes e.g. purposes, goals, problem-solving processes - historically and nowadays - for the different social groups.

## Pre-publication history

The pre-publication history for this paper can be accessed here:

http://www.biomedcentral.com/1472-6947/11/57/prepub
